# Fiber-modified hexon-chimeric oncolytic adenovirus targeting cancer associated fibroblasts inhibits tumor growth in gastric carcinoma

**DOI:** 10.18632/oncotarget.20273

**Published:** 2017-08-16

**Authors:** Tao Pang, Xinghua Wang, Jun Gao, Wei Chen, Xiao Jun Shen, Ming Ming Nie, Tianhang Luo, Kai Yin, Guoen Fang, Kai Xuan Wang, Xu Chao Xue

**Affiliations:** ^1^ Department of Gastrointestinal Surgery, ChangHai Hospital, Second Military Medical University, ShangHai, China; ^2^ Department of Microbiology, Second Military Medical University, ShangHai, China; ^3^ Department of Gastroenterology, ChangHai Hospital, Second Military Medical University, ShangHai, China; ^4^ Department of Cardiology, ChangZheng Hospital, Second Military Medical University, ShangHai, China

**Keywords:** recombinant oncolytic adenovirus, cancer associated fibroblasts, fibroblast activation protein, stromal-derived factor 1, gastric carcinoma

## Abstract

**Objective:**

To evaluate the effects of fiber-modified hexon-chimeric recombinant oncolytic adenovirus targeting cancer associated fibroblasts (CAFs) on the gastric CAFs and the transplantation tumor mice model of gastric carcinoma (GC).

**Results:**

Compared with BJ cells and GPFs, the reproduction and infectivity of P9, P9-4C or GP adenoviruses were markedly higher in gastric CAFs. In addition, P9, P9-4C or GP had a significantly relatively more killing effect on gastric CAFs compared with GPFs, and have less oncolytic effect in BJ cells. Furthermore, in transplantation tumor mice model of GC we found significantly higher hexon protein expression in tumor tissues, more decreasing tumor growth and increasing inhibitory rates after treatment of P9, P9-4C or GP adenoviruses compared with Ad adenovirus.

**Materials and Methods:**

Based on the construction of the recombinant oncolytic adenoviruses pRCAdHVR48-SDF1p-Ad/EGFP (Ad, as control) with the E1A gene transcription regulated by stromal-derived factor 1 (SDF1) promoter and the hexon replaced by hexon-chimeric (H5HVR48) gene, three fiber-modified hexon-chimeric oncolytic adenovirus through the modification fiber protein by insertion of different short peptides specifically binding to fibroblast activation protein (FAP), including pRCAdHVR48-SDF1p-FAP-P9/EGFP (P9), pRCAdHVR48-SDF1p-FAP-P9-4C/EGFP (P9-4C), pRCAdHVR48-SDF1p-FAP-GP/EGFP (GP), and their corresponding replication-defective adenovirus in parallel were reconstructed. Then the reproduction, infectivity and killing ability of the four above recombinant adenoviruses were evaluated in gastric CAFs compared with gastric para-mucosa fibroblasts (GPFs) and neonatal human foreskin fibroblasts (BJ). Furthermore, transplantation tumor mice model of GC was established, and then treated by the four above recombinant adenoviruses. Tumor size and tumor growth inhibitory rates were calculated, and histomorphology by HE staining and hexon expressions by immunohistochemistry were evaluated in tumor tissues.

**Conclusions:**

The fiber-modified hexon-chimeric recombinant oncolytic adenovirus targeting CAFs can relatively specifically kill gastric CAFs and inhibit GC cells growth *in vivo*.

## INTRODUCTION

Gastric carcinoma (GC), as a prevalent malignant disease in digestive system worldwide, is the second leading cause of cancer-related death [1]. Approximately 1 million newly diagnosed GC and 750,000 deaths caused by GC are reported each year worldwide [1, 2]. Previous study has shown long-term survival in early stage GC, while poor prognosis is frequently occurred in advanced stage GC [3]. Although more efforts have made to develop effective diagnosis and treatment methods, clinical benefits and survival are still unsatisfactory due to delayed diagnosis and lack of effective treatments [3, 4]. Therefore, it is imperative to make great effort to search for effective therapeutic treatments in GC.

Recombinant adenovirus vectors, especially oncolytic adenovirus, have been widely applied in gene therapy in cancers [5]. Previous studies have shown that oncolytic adenovirus have an anti-tumor efficacy by inducing antitumor immune responses and autophagy in cancer cells [6, 7]. Oncolytic adenovirus can induce cell death by viral lytic property, then virus reproduction contributes to the migration of the virus, thereby further amplifying anti-tumor efficacy [8]. Unfortunately, adenovirus vectors can induce the potent immunogenic toxicities, then inhibit the expression of transgene mediated by adenovirus vectors [9], which limited the extensive application in cancer therapy. Therefore, modifying the adenovirus vector is necessary to inhibit immunogenic toxicities in gene therapy.

Tumor stroma, including extracellular matrix, endothelial cells, immune cells and cancer associated fibroblasts (CAFs), is a key microenvironment of tumor growth, invasion and metastasis [9]. CAFs, as the major component in stroma, can not only contribute to tumor growth and metastasis by secreting cytokines, growth factors and adhesion molecules, but also promote tumor cells escaping from immune system and enhance resistance to radiotherapy and chemotherapy [10, 11]. Interestingly, there are two specific features of CAFs, highly expressed fibroblast activation protein (FAP) and stromal-derived factor 1 (SDF1, also known as CXCL12), which is different from normal fibroblasts [12, 13]. It has been reported that FAP possess serine protease activity and is positively correlated to tumor malignancy [14, 15]. In addition, FAP is only detected in activated fibroblasts but not normal tissues and cells [14]. SDF1 and its specific receptors (CXCR4, CXCR7) play a very important role in tumor cell proliferation, activation, neovascularization and metastasis [16]. Many studies have focused on CAFs targeted therapy, which may a very promising strategy for cancer therapy [17–19]. However, few studies have investigated the CAFs targeted therapy via oncolytic adenovirus vectors.

In the present study, three oncolytic adenovirus vectors targeting FAP through the modification fiber protein by insertion of different short peptides specifically binding to FAP were reconstructed. Their specific and effective ability of killing gastric CAFs cells were tested and verified *in vitro* and *in vivo*.

## RESULTS

### Identification of gastric CAFs

**Figure 1:**
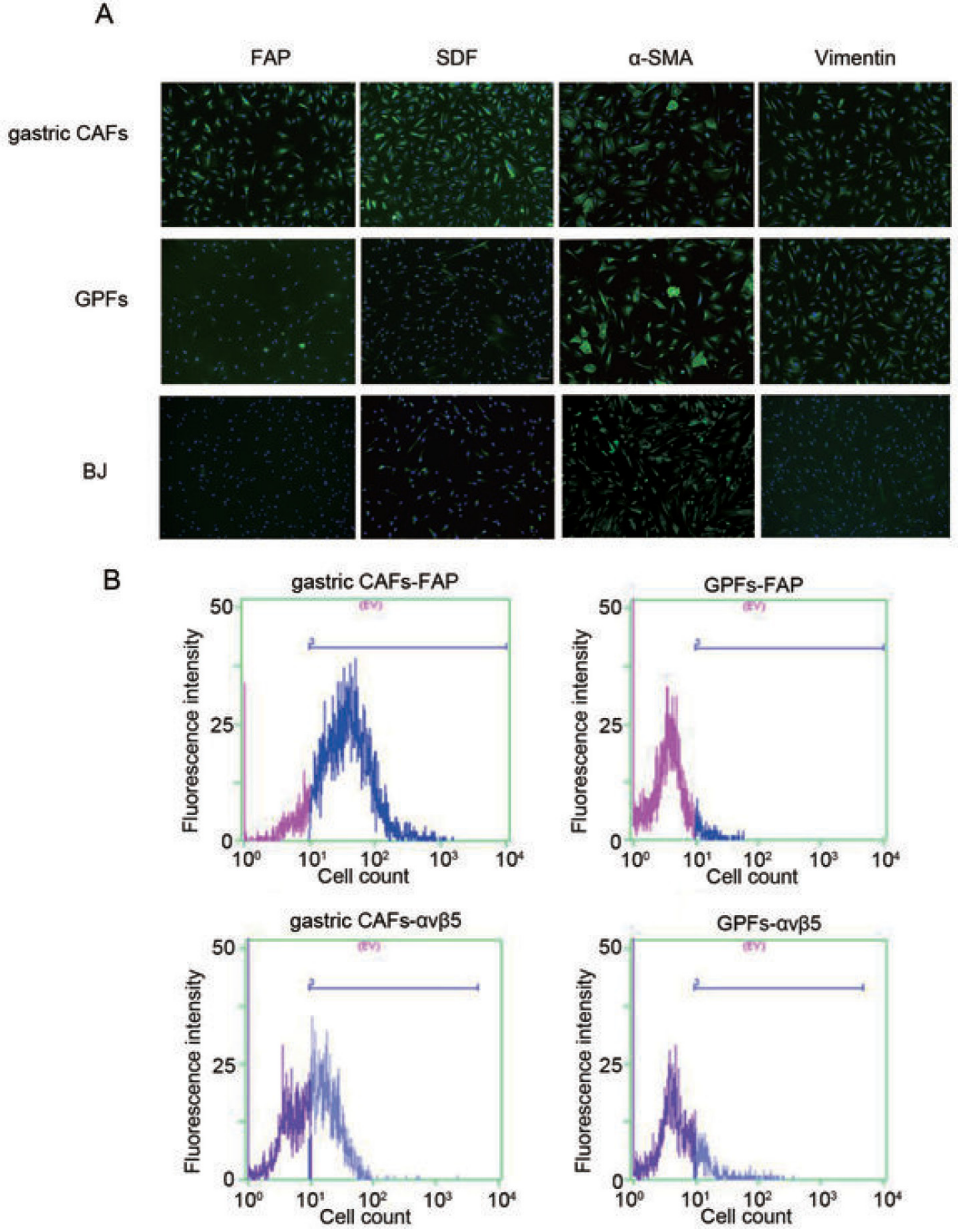
Identification of the gastric cancer associated fibroblasts (gastric CAFs), gastric para-mucosa fibroblasts (GPFs) and neonatal human foreskin fibroblasts (BJ) isolated from the GC tissue and para-carcinoma tissue respectively (**A**) The expressions of α-SMA, SDF-1, Vimentin and FAP in gastric CAFs, GPFs and BJ were detected by immunofluorescence; (**B**) The expressions of FAP and integrin αvβ5 in gastric CAFs and GPFs were detected by flow cytometry detection.

Cell immunofluorescence results showed that the gastric CAFs were positive for α-smooth muscle actin (α-SMA), stromal-derived factor 1 (SDF-1), Vimentin and FAP, while the expression of FAP and SDF-1 were higher in gastric CAFs than GPFs and BJ (Figure 1A). Also, flow cytometry results revealed that the expressions of FAP and integrin αvβ5 were obviously higher in gastric CAFs than GPFs (Figure 1B). The results indicated that FAP and SDF-1 might specifically express in gastric CAFs but not in GPFs.

### Reproduction of hexon-chimeric oncolytic adenovirus in gastric CAFs

**Figure 2:**
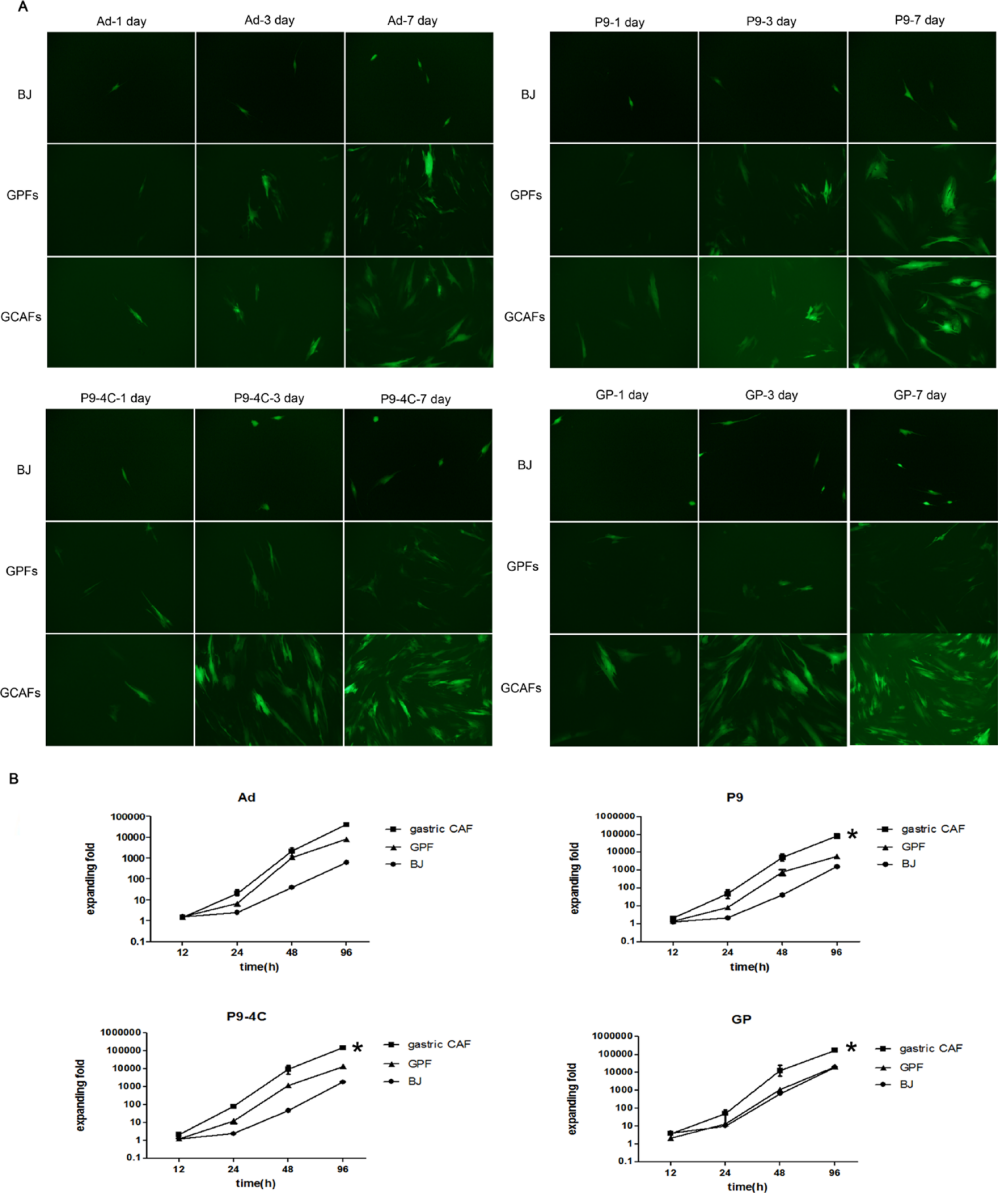
Comparison for the reproductions of four hexon-chimeric oncolytic adenovirus (P9, P9-4C, GP and Ad) in BJ cells, GPFs and gastric CAFs (**A**) The fluorescent images of the EGFP expression at 1, 3 and 7 days after the infection (MOI = 5). (**B**) The reproduction fold changes at 0, 12, 24, 48 and 96 h after the infection (MOI = 5). *, VS. GPFs, *p* < 0.05.

As shown in Figure 2A, although the reproduction fold changes of the four hexon-chimeric oncolytic adenoviruses (P9, P9-4C, GP and Ad) were all significantly increased in a time-dependent manner in BJ, GPFs and gastric CAFs cells, the three fiber-modified hexon-chimeric oncolytic adenovirus (P9, P9-4C and GP) had the most significantly expanding fold changes in gastric CAFs compared with BJ and GPFs cells. In addition, fluorescent images showed that there was almost no significant difference in the EGFP fluorescent expression of the four hexon-chimeric oncolytic adenovirus (P9, P9-4C, GP and Ad) at different infected times (1, 3 and 7 days) in BJ and GPFs cells while the EGFP fluorescent expression of three fiber-modified hexon-chimeric oncolytic adenovirus (P9, P9-4C and GP) was obviously higher in gastric CAFs cells than BJ cells (Figure 2B). The data indicated that three fiber-modified hexon-chimeric oncolytic adenovirus (P9, P9-4C and GP) specifically possessed the reproduction ability in gastric CAFs cells.

### Infectivity of hexon-chimeric oncolytic adenovirus in gastric CAFs

**Figure 3:**
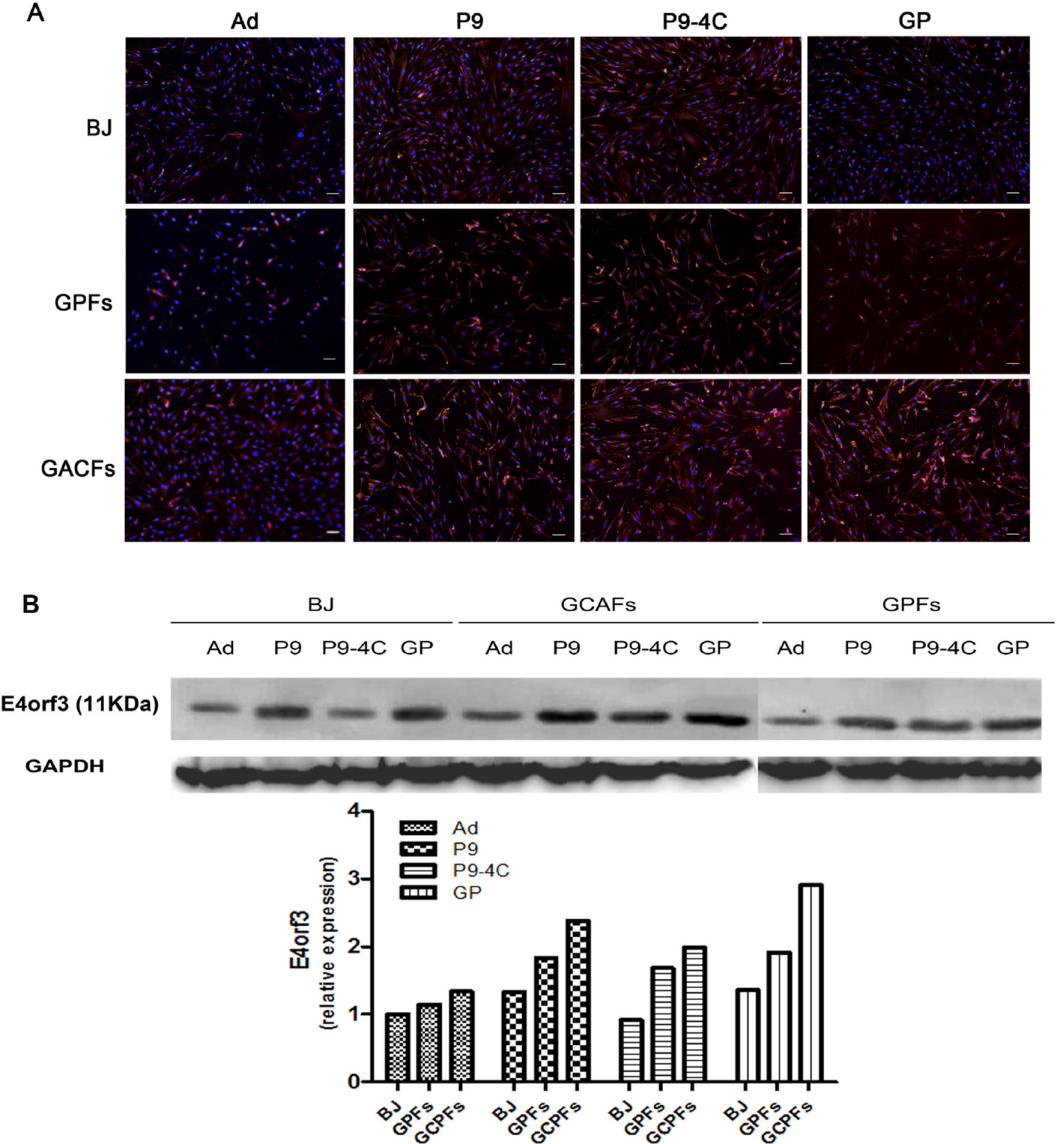
Comparison for the infectivities of four corresponding replication-defective adenovirus (P9/RFP, P9-4C/RFP, GP/RFP and Ad/RFP) in BJ cells, GPFs and gastric CAFs at 48 h after infection (MOI = 100) (**A**) The RFP expression by fluorescent images. (**B**) The expression of E4orf3 protein (11KDa) by western blotting.

After infected with the corresponding four replication-defective adenovirus, the binding capacities of adenovirus were evaluated though the observation the expression of RFP fluorescent. As shown in Figure 3A, the RFP fluorescent expressions were significantly higher in gastric CAFs cells compared with BJ and GPFs cells after infection of replication-defective adenovirus. In addition, western blotting results also showed increased expression of E4orf3 protein (11KDa) in gastric CAFs compared with BJ cells and GPFs (Figure 3B). These results indicated that the three fiber-modified hexon-chimeric oncolytic adenovirus (P9, P9-4C and GP) specifically possessed the infectivity ability in gastric CAFs cells.

### Effect of hexon-chimeric oncolytic adenovirus on gastric CAFs proliferation

**Figure 4:**
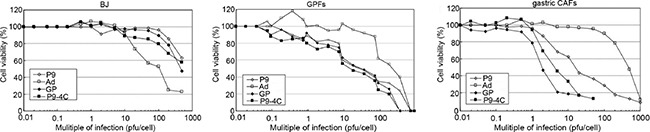
Comparison for the killing abilities of four hexon-chimeric oncolytic adenovirus (P9, P9-4C, GP and Ad) in gastric CAFs, GPFs and BJ dependently on a series of MOI (0-500) The three fiber-modified hexon-chimeric oncolytic adenovirus (P9, P9-4C and GP) effectively inhibited the viability of gastric CAFs cells than GPFs and BJ cells at MOI = 1 initially and at MOI = 10 markedly, and without obviously effect on BJ cells at less than or equal to MOI = 10.

As shown in Figure 4, in the gastric CAFs the infections of the three fiber-modified hexon-chimeric oncolytic adenovirus (P9, P9-4C and GP) could cause the reduction of cell viability obviously initially at MOI = 1 and significantly markedly at MOI = 10 compared with Ad control, while in the GPFs the infections could cause moderate reduction and in the BJ cells could cause almost not reduction. These results indicated that the three fiber-modified hexon-chimeric oncolytic adenoviruses (P9, P9-4C and GP) could specifically kill the gastric CAFs cells relatively to GPFs, and not to BJ.

### Effect of Hexon-chimeric oncolytic adenovirus on transplantation tumor mice model of GC

**Figure 5:**
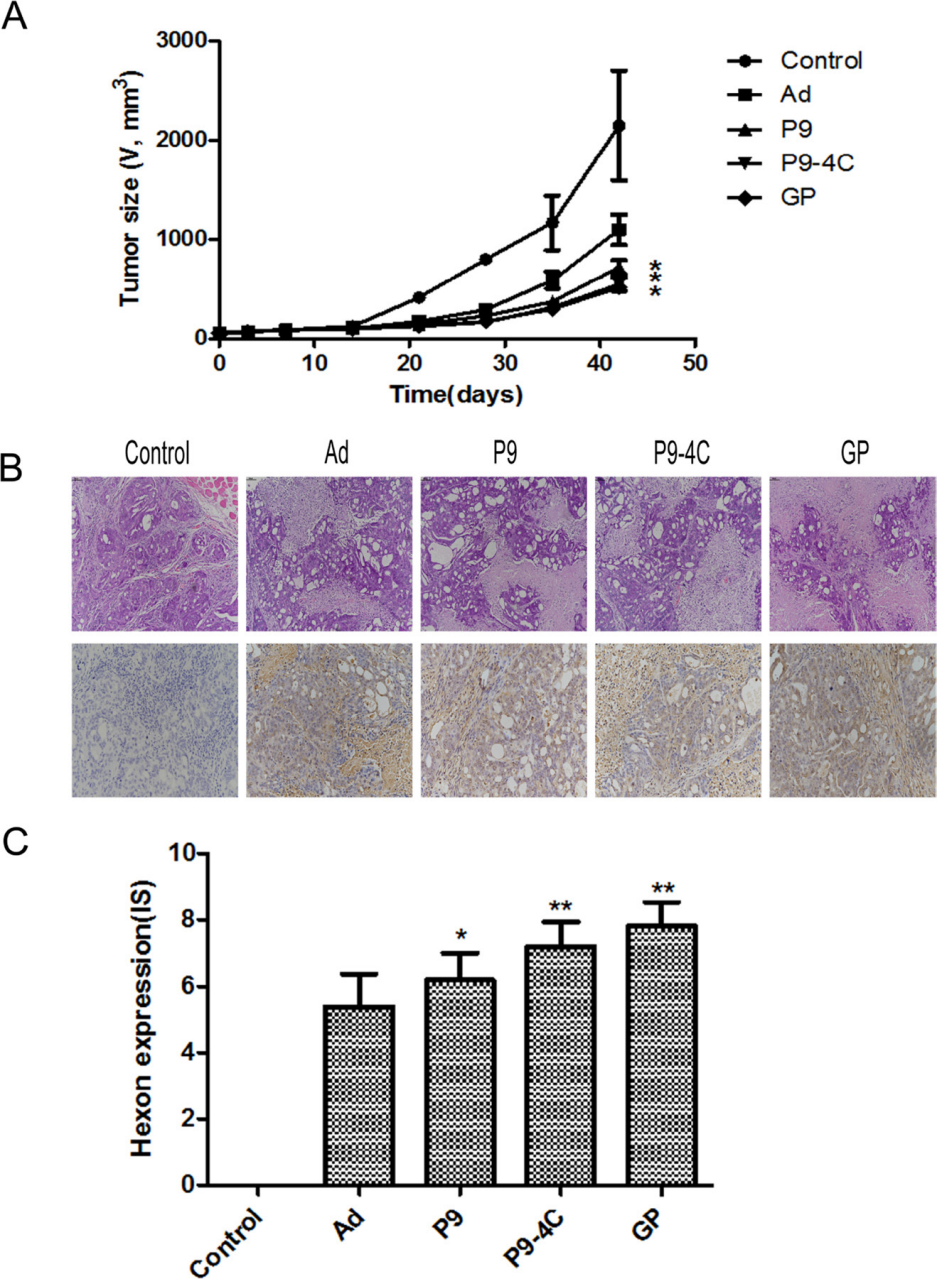
Compares for the effect of four hexon-chimeric oncolytic adenovirus (P9, P9-4C, GP and Ad) on transplantation tumor mice model of GC (**A**) The tumor growth curves in P9, P9-4C and GP groups. (**B**) The typical tumor tissue morphology by HE staining and the hexon protein expression by immunohistochemistry in P9, P9-4C and GP groups. (**C**) Compare of the IS for the expression of Hexon in tumor tissues in P9, P9-4C and GP groups. *VS. Ad group, *p* < 0.05. **VS. Ad group, *p* < 0.01.

As shown in Figure 5A, after the administration of recombinant oncolytic adenoviruses the tumor growth in P9, P9-4C and GP groups were significantly decreased compared with Ad and PBS group, and the tumor growth inhibitory rates were 50.39%, 68.66%, 76.66% and 78.10% after treatment for 42 days in Ad, P9, P9-4C, and GP groups, respectively. No obvious difference was observed in tumor size between P9 and P9-4C groups or P9-4C and GP groups at end time point 42 days. HE staining showed that tumor cells showed a lot of necrosis in Ad, P9, P9-4C, and GP groups, while tumor cells grew normally in the control group (Figure 5B). Furthermore, positive expression of hexon was not found in the control group, while the expression of hexon was higher in P9,P9-4C and GP groups than Ad groups (Figure 5B and 5C).

## DISCUSSION

In this study, three fiber-modified hexon-chimeric oncolytic adenoviral vectors, including pRCAdHVR48-SDF1p-Ad/EGFP, pRCAdHVR48-SDF1p-FAP-P9/EGFP, pRCAdHVR48-SDF1p-FAP-P9-4C/EGFP and pRCAdHVR48-SDF1p-FAP-GP/EGFP were successfully reconstructed. Compared with BJ cells and GPFs, the reproductions and infectivities of the three fiber-modified hexon-chimeric oncolytic adenoviruses were markedly higher in gastric CAFs. In addition, after infected with pRCAdHVR48-SDF1p-FAP-P9/EGFP, pRCAdHVR48-SDF1p-FAP-P9-4C/EGFP, pRCAdHVR48-SDF1p-FAP-GP/EGFP adenovirus, cell viability was significantly decreased in gastric CAFs compared with pRCAdHVR48-SDF1p-Ad/EGFP adenovirus. However, BJ cells showed no significant change of the cell viability when infected with pRCAdHVR48-SDF1p-FAP-P9/EGFP, pRCAdHVR48-SDF1p-FAP-P9-4C/EGFP or pRCAdHVR48-SDF1p-FAP-GP/EGFP adenovirus compared with pRCAdHVR48-SDF1p-Ad/EGFP adenovirus, indicating the safety of fiber-modified hexon-chimeric oncolytic adenoviral vectors in normal cells. Furthermore, pRCAdHVR48-SDF1p-FAP-P9/EGFP, pRCAdHVR48-SDF1p-FAP-P9-4C/EGFP or pRCAdHVR48-SDF1p-FAP-GP/EGFP adenovirus could inhibit tumor growth in transplantation tumor mice model of GC.

Increasing studies had shown the effective therapy of recombinant oncolytic adenovirus vectors in cancers [5]. Lucas *et al.* [20] had reported that hexon-modified oncolytic adenoviral vectors targeting TGF-β receptor CKS17 had increased reproduction and safety, and could effectively inhibited tumor growth in pancreatic cancer. Previous study showed that original Ad5 hexon gene in hexon-chimeric adenoviral vectors was replaced by H5HVR48, which could reduce virus immunogenicity and hepatotoxicity [21]. Zhang et al. [22] also reported that Ad5 hexon substituted with H5HVR48 could induced less liver toxicity and increased antitumor response in breast cancer. Similarly, in this study, three fiber-modified hexon-chimeric oncolytic adenoviral vectors contained Ad48 hypervariable region and these adenovirus had less effect on the reproduction, infectivity and cell viability in BJ cells and GPFs. These results indicated a satisfactory security of fiber-modified hexon-chimeric oncolytic adenovirus in normal epithelial cell and fibroblasts.

It was well-known that CAFs was closely associated with tumor genesis, development, recidivation and metastasis [23]. Previous studies had shown that adenoviral E1A gene regulated by the promoters of prostate specific antigen and α fetal protein could effectively and specifically inhibited tumor growth in prostatic cancer and hepatocellular carcinoma [24, 25]. In addition, Kasuya *et al.* [26] also reported that adenoviral E1 gene controlled by mucoprotein 1 promoter could selectively induce reproduction and infectivity of adenovirus in breast cancer. It had been demonstrated that over-expressed SDF1 in CAFs contributed to tumor growth and angiogenesis [27, 28]. Therefore, in this study, the expression of E1A gene was regulated by SDF1 promoter, which might increase the specificity of fiber-modified hexon-chimeric oncolytic adenovirus in gastric CAFs. Meanwhile, E1B gene was controlled by HER that was DNA-binding site of HIF. Up-regulated HIF was associated with tumor hypoxic microenvironment, and could induce tumor growth. The dual regulation of SDF1 and HER might further increase the reproduction and infectivity of oncolytic adenovirus. Consistently, the present study showed increased reproduction and infectivity of three fiber-modified hexon-chimeric oncolytic adenoviral vectors in gastric CAFs. FAP was also specifically expressed in CAFs [14, 29] and was associated with cell invasion, growth and angiogenesis in tumors [30]. In this study, specific FAP recognition short peptides (P9, P9-4C and GP) were inserted into oncolytic adenoviral vectors in this study, which might further increased the specificity of fiber-modified hexon-chimeric oncolytic adenoviral vectors in gastric GAFs. Actually, this study also revealed that the fiber-modified hexon-chimeric oncolytic adenovirus had increased capable of killing gastric CAFs compared with oncolytic adenovirus without the fiber-modified by FAP recognition short peptides. All these results indicated that fiber-modified hexon-chimeric oncolytic adenovirus could replicate in gastric CAFs and effectively and specifically induce the death of gastric CAFs.

In addition, *in vivo* experiments of this study showed positive expression of hexon in mice infected with the four oncolytic adenovirus via tail vein injection, and the higher expression level in P9, P9-4C and GP groups than Ad groups, confirming the activity of fiber-modified hexon-chimeric oncolytic adenovirus *in vivo*. Many studies had reported the application of oncolytic adenovirus *in vivo* and reconstructed oncolytic adenovirus could effectively inhibited tumor growth with less virus immunogenicity and hepatotoxicity in animal and clinical experiments [6, 31, 32]. Consistently, we also revealed that fiber-modified hexon-chimeric oncolytic adenovirus inhibited tumor growth in transplantation tumor mice model of GC, and specifically possessed the killing ability for gastric CAFs cells, indicating the their anti-tumor effect for potential clinical application.

## MATERIALS AND METHODS

### Cell lines

Human GC cell line SGC-7901 was obtained from Changhai Hospital (Shanghai, China). Human embryonic kidney cell line HEK293 was purchased from Microbix Biosystems, Inc. (Toronto, Canada). Neonatal human foreskin fibroblasts BJ were provided by Dr. Su Changqing from Eastern Hepatobiliary hospital (Shanghai, China). All these cells were cultured in Dulbecco's Modified Eagle Media (DMEM, Gibco, GrandIsland, NY, USA) containing 10% fetal bovine serum (FBS).

### Isolation and culture of primary cells

GC tissue and para-carcinoma tissue were collected from 5 patients with GC underwent operation therapy in first general surgery department, Changhai hospital from April to August in 2012. The tissues were sliced into 1–2 mm^3^ size of tissue mass, and immersed in sterile D-Hank's solution containing 100 U/mL penicillin, 100 mg/mL streptomycin and 2 μg/mL amphotericin B for 10 min. After filtration with 100 μm strainer, tissues were digested in DMEM/F12 containing collagenase I (2 mg/mL) and hyaluronidase (250 U/mL) for 2 h at 37°C, and then centrifuged at 2000 rpm for 10 min. The isolated gastric CAFs and gastric para-mucosa fibroblasts (GPFs) were cultured in DMEM/F12 containing 10% FBS. Gastric CAFs and GPFs were identified using cell immunofluorescence and flow cytometry detection.

### Cell immunofluorescence

Gastric CAFs, GPFs and BJ cells were fixed with 4% pre-cooled paraformaldehyde for 15 min, followed by blocked by 1% goat serum at room temperature for 1 h. The gastric CAFs were then incubated at 4°C overnight with anti-α-smooth muscle actin (α-SMA, MAB1420; R&D, Minneapolis, MN, USA), SDF-1 (MAB350; R&D), FAP (ab207178; Abcam, Cambridge, MA, USA), Vimentin (sc-73258; Santa Cruz, Santa Cruz, CA, USA) monoclonal antibodies at a dilution of 1:100. After washed with PBS, the cells were incubated with Alexa Fluor labeled IgG (H+L) second antibody (A21202; Invitrogen) at a dilution of 1:200 at room temperature for 1 h. DAPI was added for 5 min to stain cell nucleus. The cells were observed in inverted fluorescence microscope (Olympus, Tokyo, Japan).

### Flow cytometry detection

Totally, 1 × 10^6^ cells/mL gastric CAFs or GPFs were stained with anti-FAP (ab207178; Abcam) and integrin αvβ5 (MAB2528; R&D) monoclonal antibodies (dilution ratio of 1:50, BD, USA) for 30 min under 4°C and dark condition, respectively. After washed with PBS, the cells were detected using FACSCaliburTM (BD, San Diego, CA, USA) and observed under fluorescence microscopy (Olympus, Tokyo, Japan).

### Preparation of adenoviral vectors and viruses

The process of the adenovirus reconstruction was illustrated in Supplementary Figure 1, and was basically described as follows. The p-ENTR-GF-LA vector, pShE1 vector and pAdRc/delLA vector were provided by Professor Qian Qijun from Eastern Hepatobiliary hospital. The E1A gene promoter (TERTp) in the pGS502 shuttle vector (constructed by our team) was substituted by SDF1 (CXCL12) promoter (synthesized by Huada Genomic Biotechnology Co., Ltd, Shanghai, China). Meanwhile, E1B gene promoter (5 × HER and CMV) was reserved to construct a new shuttle vector pShSDF1p-E1. Three base sequences corresponding to specific FAP recognition peptide (P9,TSGPNQEQKTSLLLLVWP; P9-4C, TSCDCTSGPNQEQKCFC; GP, TSGPTSWP) was inserted into SpeI site of pENTR-GF-LA using In-Fusion site-specific recombination technology, and then EGFP gene was cloned into pENTR-GF-LA to obtain pENTR/FAP/EGFP. Original Ad5 hexon gene in the pAdRc/delLA vector was replaced by hexon-chimeric gene (H5HVR48), which was named as hexon-chimeric adenoviral plasmid vector pAd5HVR48-Rc/delLA containing Ad48 hypervariable region. Then pENTR/FAP/EGFP and pAd5HVR48-Rc/delLA were recombined using high-performance site-specific recombination technology (Gateway). The recon was selected using ccdB toxicity and ampicillin resistance, and right terminal sequence of reconstructed adenovirus genome was inserted back into plasmid vector, which was named as fiber modified hexon-chimeric recombination adenovirus skeleton vector pAd5HVR48/FAP-EGFP/delE1E3. The recombination adenovirus plasmid pAd5HVR48/SDFp/FAP/EGFP was generated by the co-transfection of shuttle vector pShSDF1p-E1 and skeleton vector pAd5HVR48/FAP/delE1E3 into E.coli DH5α competent cell. Inverted terminal repeat (ITR) sequence in adenoviral genome terminals was exposed after fully digestion of verified pAd5HVR48/SDFp/FAP/EGFP plasmid by restriction enzyme PacI. Then the recombination adenovirus recon was transfected into HEK293A cells by Lipofectamine 2000 (Invitrogen, Carlsbad, CA, USA). The fiber-modified hexon-chimeric oncolytic adenovirus, including pRCAdHVR48-SDF1p-FAP-P9/EGFP (P9), pRCAdHVR48-SDF1p-FAP-P9-4C/EGFP (P9-4C), pRCAdHVR48-SDF1p-FAP-GP/EGFP (GP) and pRCAdHVR48-SDF1p-Ad/EGFP (Ad, without fiber-modified as control) were obtained by plaque purification for three times. In parallelly, their corresponding replication-defective adenovirus in which the EGFP gene was replaced by RFP gene were reconstructed through only the skeleton vector transfected into HEK293A cells. Virus titer was measured by 50% tissue culture infectious doses (TCID50) and purified adenoviruses were finally stored in −80°C for future utilization.

### Virus reproduction assay

Cells in logarithmic phase were seeded into six well plates (1 × 10^5^ each well) and cultured in 37°C, 5% CO_2_ atmosphere for 24 h. Then, BJ cells, gastric CAFs and GPFs were cultured in serum-free medium and infected with four recombinant adenovirus, including pRCAdHVR48-SDF1p-Ad/EGFP, pRCAdHVR48-SDF1p-P9/EGFP, pRCAdHVR48-SDF1p-P9-4C/EGFP and pRCAdHVR48-SDF1p-GP/EGFP, with a multiplicity of infection (MOI) of 5, respectively. After cultured for 2 h, medium was changed with 5% blood serum medium. Cells and supernatant at 0, 6, 12, 48 and 96 h were collected after virus infection. The fold changes of virus reproduction were calculated using the titers at different time points by TCID50 method.

### Virus infectivity detection

Gastric CAFs, GPFs and BJ cells in logarithmic phase were seeded into six well plates (1 × 10^5^ each well) for 24 h. Then, the cells were infected with four corresponding replication-defective adenovirus, including pRCAdHVR48-FAP-P9/RFP (P9/RFP), pRCAdHVR48-FAP-P9-4C/RFP (P9-4C/RFP), pRCAdHVR48-FAP-GP/RFP (GP/RFP) and pRCAdHVR48-Ad/RFP (Ad/RFP), with a MOI of 100, respectively, at 4°C for 1 h. Virus not binding with cells were washed by cold PBS. Then cells were cultured in 5% DMEM/F12 medium at 37°C, 5% CO_2_ for 48 h, and observed under fluorescence microscope. Lastly, the gastric CAFs were collected to detect the expression of E4orf3 protein using Western blotting.

### Western blotting

The infected cells were collected and placed on ice for 30 min in RIPA lysis buffer (Pierce, Rockford, IL, USA). Protein concentration was detected by the BCA Protein Quantitative Assay (Shanghai Sangon Biotech Co., Ltd, China). Then protein sample was separated on SDS-PAGE gel, and then blotted onto nitrocellulose membranes (Mippore, Billerica, MA, USA). After blocked in 5% nonfat milk, the membranes were incubated overnight at 4°C with anti-E4 rabbit polyclonal antibody (kindly provided by Professor Qian Qijun) and GAPDH monoclonal antibody (1:2000, WB0197; Shanghai Weiao Biotechnology co., LTD, China). After washed for three times with PBS, the membranes were incubated with IgG(H+L)-HRP second antibody (1:5000, Santa Cruz) for 2 h at room temperature. Ultimately, the proteins were detected with Enhanced chemiluminescence (Millipore).

### Cell killing ability detection

Gastric CAFs, GPFs and BJ cells in logarithmic phase were seeded into 96-well plates (1 × 10^4^ each well) for 24 h. Then, the cells were infected with four oncolytic adenovirus, including P9, P9-4C, GP and Ad with a MOI of 0.01, 0.1, 1, 10 and 100, respectively. After infection for 7 days, WST1 (10 mL/well) was added in the cells for 2 h at 37°C. Ultimately, the absorbances were read at 450 nm using a microplate reader (Molecular Devices, USA).

### Animal models and treatments

Totally, 20 BALB/C nude mice (half of male and female) were purchased from Shanghai slack laboratory animal Co., LTD (China). All mice were acclimatized for 7 days before the experiments. To develop a transplantation tumor model of GC, human gastric cancer cell MKN-45 (1 × 10^8^ each mice) was subcutaneous inoculated into the right back of nude mice for 18 days. Then, mice were randomly assigned into 5 groups with 4 mice (2 male and 2 female) in each group: Control (PBS), Ad, P9, P9-4C and GP groups. The adenovirus (2 × 10^8^pfu in every time for one animal) or 500 μL PBS (as control) were administrated via tail vein injection every other day for 5 days. Tumor size (the longest and the shortest diameter) of mice in each group were measured after treatment for 0, 3, 7, 14, 21, 28, 35 and 42 days. Tumor size (V, mm^3^) was calculated as the longest diameter × the shortest diameter^2^/2. Tumor growth inhibitory rate was calculated as [1 – (V_detected time_ – V_initial time_)/(V_detected time_ – V_initial time_)] × 100%. The mice were sacrificed at 42 days after treatment, and the tumor tissues were collected for the following experiments.

### Hematoxylin and eosin (H&E) staining

Tumor tissues were fixed in 4% paraformaldehyde for 24 h. After paraffin imbedding, the tissues were sliced into 4 μm-thick sections. The sections were dehydrated with gradient ethanol, and then stained with hematoxylin for 5 min. After differentiated in 1% hydrochloric acid alcohol for 2s, the sections were then incubated in ammonia water, followed by the staining with eosin. Ultimately, the sections were dehydrated, cleared, mounted with neutral resin, and observed using light microscopy (Olympus, Japan).

### Immunohistochemistry (IHC) for hexon

Tumor tissues were fixed in 4% paraformaldehyde. Paraffin sections (4 μm-thick) of tissues were dewaxed and dehydrated in xylene and gradient ethanol, respectively. Heated-citrate buffer solution (PH 6.0) was used for antigen retrieval. Endogenous peroxidase activity was carried out using 3% hydrogen peroxide solution for 10 min at room temperature. Next, sections were blocked with 1% serum, and then incubated with rabbit anti-Hexon polyclonal antibody (1:400, ab8251; Abcam) at 4°C overnight. PBS was used as the primary antibody in the negative controls. After washed with PBS, sections were incubated with goat anti-rabbit IgG-HRP secondary antibody (1:500, 111-035-045; Jackson ImmunoResearch Laboratories Inc., West Grove, PA, USA) for 45 min, and then stained with diaminobenzidine. Lastly, all sections were re-stained with hematoxylin, dehydrated and sealed. Sections were observed under a light microscope (Nikon, Japan) and positive staining was defined as brown particles in the cytomembrane or cytoplasm.

The immunoreactive score (IS) was applied to evaluate both the intensity of immunohistochemical stain and the proportion of the stained cells (cytomembrane, cytoplasm and/or nuclei) by counting minimum 200 cells per field at 40× magnification in 5 randomly selected fields. The staining intensity was graded as follows: 0, negative; 1, weak; 2, moderate; 3, strong. The positive cells were quantified as a percentage of the total number of cells and assigned to one of five categories: 0, < 5%; 1, 5–25%; 2, 26–50%; 3, 51–75%; and 4, > 75%. Two independent pathologists who were blinded to the results marked the scores. The mean scores of every sample was then compared, and if the difference between the counts and intensity by the two pathologists was less than 20% of the maximum value, the mean value was used as the final count. If the difference exceeded 20%, the reason would be discussed and the evaluation would be re-done. The percentage of positive cells was multiplied by the staining intensity to generate the IS for each specimen.

### Statistical analysis

Statistical analysis was performed by SPSS 10.0 statistical analysis software (SPSS Inc., Chicago, IL, USA). Data were expressed as the mean ± S.D. The analyses for the reproduction and the tumor growth between the differential adenviour groups were performed by GLM Repeated Measures, and the analyses for the IS between the differential adenviour groups were performed by one-way ANOVA. A value of *P* < 0.05 was considered significant.

## CONCLUSIONS

The present study reveals that recombinant adenovirus targeting CAFs can specifically and effectively kill gastric CAFs and inhibit GC cells growth *in vivo*, indicates that fiber-modified hexon-chimeric oncolytic adenovirus targeting CAFs may be a safe and effective strategy for GC targeted therapy.

## SUPPLEMENTARY MATERIALS AND FIGURES


