# Supplementary material for: Fiber-modified hexon-chimeric oncolytic adenovirus targeting cancer associated fibroblasts inhibits tumor growth in gastric carcinoma
Source: Oncotarget. 2017 Aug 16;8(44):76468–78. doi: 10.18632/oncotarget.20273 (PMC5652720; doi:10.18632/oncotarget.20273)
Supplement: Supplementary file 1 [file oncotarget-08-76468-s001.pdf]

Fiber-modified hexon-chimeric oncolytic adenovirus targeting cancer associated fibroblasts inhibits tumor growth in gastric carcinoma

SUPPLEMENTARY MATERIALS

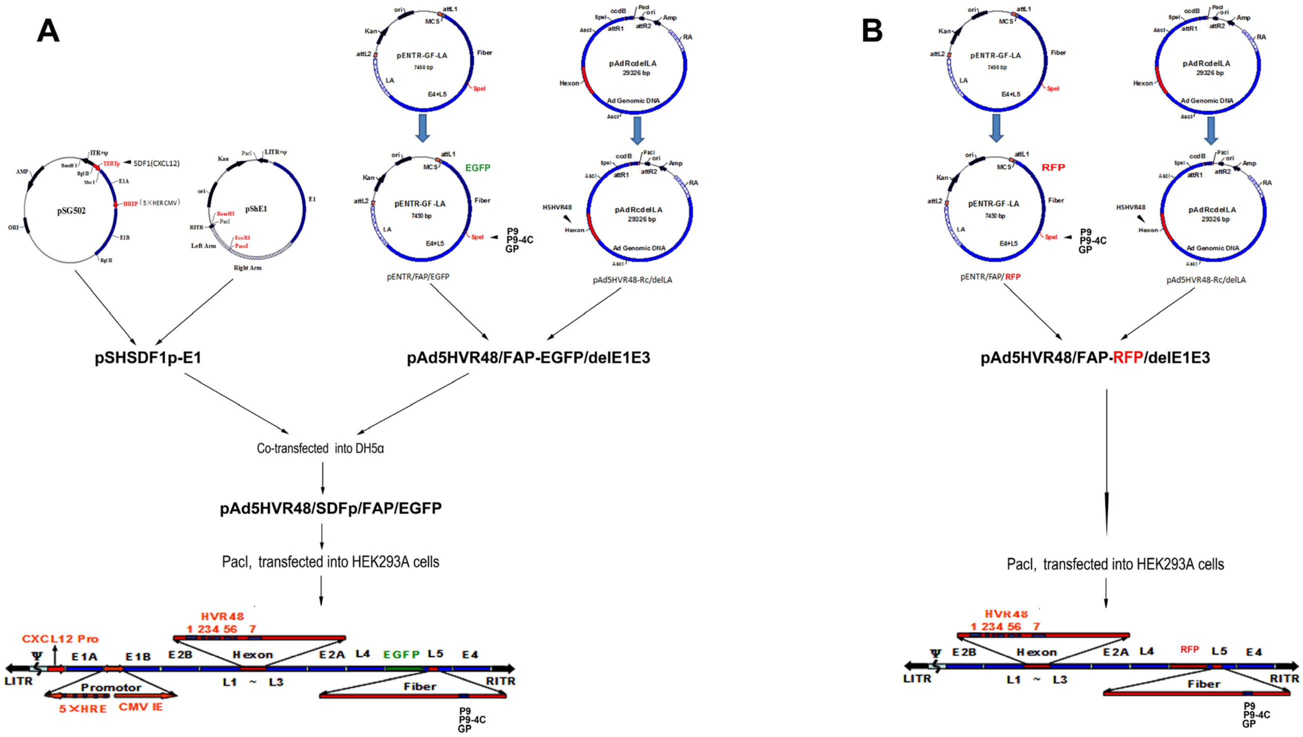

Supplementary Figure 1: Illustration for the reconstruction process of the four hexon-chimeric oncolytic adenovirus (P9, P9-4C, GP and Ad) adenoviruses (**A**) and their corresponding replication-defective adenovirus (**B**).
